# South Yorkshire Cohort: a 'cohort trials facility' study of health and weight - Protocol for the recruitment phase

**DOI:** 10.1186/1471-2458-11-640

**Published:** 2011-08-11

**Authors:** Clare Relton, Paul Bissell, Christine Smith, Joanna Blackburn, Cindy L Cooper, Jon Nicholl, Angela Tod, Rob Copeland, Amanda Loban, Tim Chater, Kate Thomas, Tracy Young, Carol Weir, Gill Harrison, Alison Millbourn, Rachel Manners

**Affiliations:** 1 National Institute for Health Research Collaboration for Leadership in Applied Health Research and Care for South Yorkshire (NIHR CLAHRC-SY), Sheffield Teaching Hospitals, 11 Broomfield Road, Sheffield, S10 2SE, UK; 2 School of Health and Related Research (ScHARR), University of Sheffield, 30 Regent Street, Sheffield S1 4DA, UK; 3 Research & Development Department, Barnsley Hospital NHS Foundation Trust, Gawber Road, Barnsley, S75 2EP, UK; 4 The Centre for Sport & Exercise Science, Sheffield Hallam University, Collegiate Crescent, Sheffield, S10 2BP, UK; 5 Public Health Directorate, NHS Rotherham, Oak House, Moorhead Way, Bramley, Rotherham, S66 1YY, UK; 6 Public Health, NHS Barnsley, Hilder House, 49/51 Gawber Road, Barnsley, S75 2PY, UK; 7 Public Health, NHS Doncaster, White Rose House, Ten Pound Walk, Doncaster, DN4 5DJ, UK

## Abstract

**Background:**

Growing levels of both obesity and chronic disease in the general population pose a major public health problem. In the UK, an innovative 'health and weight' cohort trials facility, the 'South Yorkshire Cohort', is being built in order to provide robust evidence to inform policy, commissioning and clinical decisions in this field. This protocol reports the design of the facility and outlines the recruitment phase methods.

**Method/Design:**

The South Yorkshire Cohort health and weight study uses the cohort multiple randomised controlled trial design. This design recruits a large observational cohort of patients with the condition(s) of interest which then provides a facility for multiple randomised controlled trials (with large representative samples of participants, long term outcomes as standard, increased comparability between each trial conducted within the cohort and increased efficiency particularly for trials of expensive interventions) as well as ongoing information as to the natural history of the condition and treatment as usual.

This study aims to recruit 20,000 participants to the population based South Yorkshire Cohort health and weight research trials facility. Participants are recruited by invitation letters from their General Practitioners. Data is collected using postal and/or online patient self completed Health Questionnaires. NHS numbers will be used to facilitate record linkage and access to routine data. Participants are eligible if they are: aged 16 - 85 years, registered with one of 40 practices in South Yorkshire, provide consent for further contact from the researchers and to have their information used to look at the benefit of health treatments. The first wave of data is being collected during 2010/12 and further waves are planned at 2 - 5 year intervals for the planned 20 year duration of the facility.

**Discussion:**

The South Yorkshire Cohort combines the strengths of the standard observational, longitudinal cohort study design with a population based cohort facility for multiple randomised controlled trials in a range of long term health and weight related conditions (including obesity). This infrastructure will allow the rapid and cheap identification and recruitment of patients, and facilitate the provision of robust evidence to inform the management and self-management of health and weight.

## Background

Growing levels of obesity and obesity related conditions in England (and much of the developed world) pose a major public health problem requiring the urgent attention of policy makers, health professionals and the public alike [1]. The recent influential Foresight [2] review of the evidence on the causes, prevention and treatment of obesity highlighted various factors germane to assembling an effective public health response to this problem. This report identified the causes of obesity as being *"embedded in an extremely complex biological system set within an equally complex societal framework" *and highlighted the impact of changes in work patterns, transport, food production, food sales and shifts in values on obesity over the past five decades. Following the evidence provided by Foresight, the Healthy Weight Healthy Lives report [1] identified the following range of requirements for a cross government research and surveillance plan for England:

• short, medium and longer term research and surveillance

• research to read across to other policy and research agendas, e.g. vascular disease, cancer, diabetes, liver disease

• evaluate policies aimed at tackling obesity including impact on health inequalities

• evaluate long term impact of interventions including assessing cost effectiveness

• research to target large population groups and evaluation of 'natural experiments'.

The study described here - the South Yorkshire Cohort study [3] - focuses on fulfilling these five requirements by building a short, medium and longer term population based research and surveillance facility for weight and health.

This study also seeks to address fundamental flaws in the methodology of existing studies which have hindered the ability to identify effective public health and clinical interventions (a core observation made in the National Institute for Health and Clinical Excellence (NICE) clinical guidelines for obesity [4].

**Figure 1:**
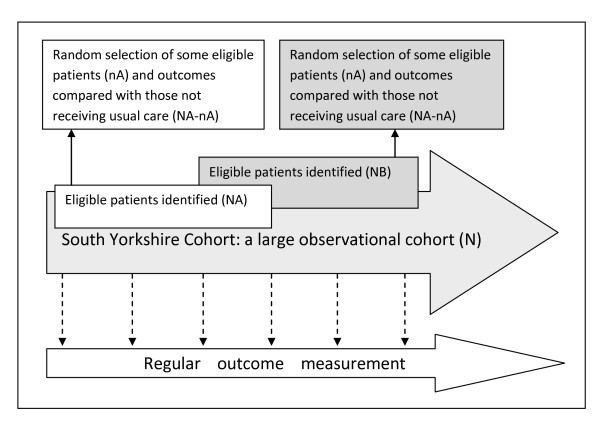
**South Yorkshire Cohort: a 'cohort trials facility' study of health and weight**.

In order to inform NHS commissioning of obesity services, a range of information is required, namely, data from longitudinal observational studies and randomised controlled trials conducted in populations representative of the 'with need' population. Although the information is needed, few interventions or services currently benefit from this level of evidence. Existing longitudinal birth cohort studies provide long term outcome data on weight and chronic disease in the UK e.g. [5,6] and many other datasets provide data on adult weight and height in the UK, e.g. Health Survey for England [7], Quality Outcomes Framework [8], QResearch [9], however, none of these studies provide all the information required by the NHS. Birth cohorts only collect data on individuals born in that particular year/month, the annual home interviews of the Health Survey for England [7] collect cross sectional (not longitudinal) data, Quality Outcomes Framework [8] collects data opportunistically from those seeking treatment (and thus is unlikely to be representative of the general population), and QResearch [9] collects GP reported but not patient reported data. The South Yorkshire Cohort differs from these existing data sources in that it will provide an infrastructure which combines the strengths of the standard observational, longitudinal cohort study design coupled with a facility for multiple randomised controlled trials (see Figure 1). Additional qualitative and observational quantitative projects will also be able to be nested within this facility.

Thus the aim of this innovative population based 'cohort trials facility' is to build research capacity across policy and research agendas, enable evaluation of policies aimed at tackling obesity including impact on health inequalities, enable the evaluation of natural experiments and the long term impact of interventions, including clinical and cost effectiveness.

### Study aims

The broad aims of the South Yorkshire Cohort are to:

• initiate a programme of research into the management and self-management of weight and long term conditions in adults in South Yorkshire

• to explore the relationship between key variables from an NHS perspective: long term chronic disease, weight change, quality of life, prescribed and over the counter medication, NHS and non NHS healthcare resource use and ethnicity and social class

• provide a cohort facility for multiple trials of interventions for the management and self- management of weight and long term chronic health conditions

• provide a research facility for longitudinal and qualitative studies in weight and long term conditions

The specific research objectives agreed as of 27^th ^June 2011 by the South Yorkshire Cohort Management Group are as follows:

1. To report the prevalence of long term health conditions and associated variables

2. To report the prevalence of obesity and overweight and associated variables

3. To describe the characteristics of weight management and self-management strategies self reported by the South Yorkshire adult population

4. To provide a detailed profile of the health and weight of underweight, normal weight, overweight, obese and morbidly obese patients registered with each participating GP practice, plus the types of services and treatments used by patients to manage their weight

5. To evaluate the clinical and cost effectiveness of potential weight management interventions for obese and overweight individuals and populations

6. To investigate the natural course of obesity and the effect of particular variables on health outcomes associated with obesity and its self-management

## Method/Design

### Study design

The South Yorkshire Cohort uses the cohort multiple randomised controlled trial (cmRCT) design [10] (see Figure 1). Key features of this design are:

(I) Recruitment of a large observational cohort of patients with condition(s) of interest

(II) Regular outcome measurement for the whole cohort

(III) Capacity for multiple RCTs over time

(IV) For each RCT, all eligible patients in the cohort are identified (NA)

(V) Random selection of some eligible patients (nA), and trial treatment offered

(VI) Outcomes of randomly selected patients (nA) are compared to usual care i.e. the outcomes of those eligible patients not randomly selected (NA - nA)

(VII) 'Patient centred' informed consent i.e. patient information and consent aim to replicate that of real world routine healthcare.

The benefits of using the cmRCT design will help address some of the shortcomings that prevent many pragmatic randomised controlled trials fulfilling their potential of giving robust evidence that clinicians can apply to their clinical populations. In comparison to randomised controlled trials, longitudinal observational studies can recruit a greater quantity of patients and more representative samples of patients. By separating consent to take part in the observational cohort from consent to 'try' a particular intervention, the staged and 'patient centred' information and consent procedures that exist in routine healthcare are replicated. This ensures a more ethical research design and one where patient preferences are minimally affected by the research design. Moreover, in comparison to the current single pragmatic trial, the use of an observational cohort provides important additional benefits:

• a multiple randomised controlled trial facility

• long term outcomes as standard

• ongoing information as to the natural history of the condition(s) and treatment as usual

• increased comparability between each trial conducted within the cohort

• increased efficiency, particularly for expensive or high risk interventions

• rapid and cheap identification and recruitment of patients

The 'cmRCT' design enables more reliable direct and indirect comparisons than is currently possible because all treatments have the same treatment as usual (i.e. standard care) comparator and use the same core outcomes [10]. Standard randomised controlled trial designs often struggle to recruit and consequently have to randomly allocate all patients using equal randomisation to maximise statistical power within their total sample size. The large numbers of patients recruited to the cohort enables trials to have increased statistical power and/or unequal randomisation resulting in either strengthened inferences and/or lower total treatment costs than standard trial designs and significant cost savings for trials of expensive treatments. Because of the large cohort, unequal randomisation can be used more easily (i.e., randomise more to the inexpensive treatment group), which improves the efficiency of trials of high cost interventions compared to equal allocation. Furthermore, information on those who decline treatment provides information as to the acceptability of the treatment and thus the generalisability of the trial results. Once the cohort is established it potentially allows for rapid and cheap identification and recruitment of patients.

The application of the cmRCT design to the question of obesity and other long term chronic conditions will enable both ongoing surveillance and evaluation of the clinical and cost effectiveness of interventions in South Yorkshire, together with a resource for research and a facility for testing interventions for obesity and other chronic conditions.

### Study setting

South Yorkshire is a metropolitan county in the Yorkshire and Humber region of England with a population of 1.29 m in three metropolitan boroughs (Barnsley, Rotherham, Doncaster), and the city of Sheffield. In 2008, a quarter of adults in England (24% of men and 25% of women aged 16 or over) were classified as obese (Body Mass Index ≥30 kg/m^2^) [11], however much of South Yorkshire has higher rates (Barnsley 28.4%, Doncaster 29%, Rotherham 27.6%, Sheffield 23.7%) [12].

### Study sample

The target population is all patients registered with GP practices in South Yorkshire aged 16 to 85 years. We use a two stage sampling method, which initially recruits GP practices and then recruits patients registered with these practices. Forty practices from South Yorkshire will be recruited and eligible patients are invited to participate in the South Yorkshire Cohort.

### Eligibility criteria

All patients registered with the recruited GP practices aged 16 to 85 years will be eligible for inclusion in the South Yorkshire Cohort study. Eligibility criteria for studies embedded within the South Yorkshire Cohort will vary.

### Sample size

Cohort studies vary considerably in size. The purpose of the South Yorkshire Cohort study is to provide a cohort facility for multiple trials and other studies, thus the cohort sample size has been calculated in order to provide amongst other things a sufficient number of overweight, obese and morbidly obese patients for a range of randomised controlled trials to be conducted. We aim to recruit a total of 20,000 adults to the South Yorkshire Cohort multiple trials facility, which will provide an estimated 6,800 overweight adults, 4,400 obese adults and 400 morbidly obese adults.

### Recruitment

Recruitment began in June 2010 and is planned to continue until November 2012. GP practices are recruited by email, letter and phone (Additional File 1). Recruited GP practices send out an invitation letter (Additional File 2) to all their eligible patients. Postage stamps rather than prepaid envelopes are used to make the envelope appear less official and more personal and thus help improve the response rate [13]. The personalised invitation letter provides the patient with information about the study. Also enclosed is the South Yorkshire Cohort 'Health Questionnaire' (Additional File 3). Patients are also offered access to an online version of the Health Questionnaire available at: http://www.syc.shef.ac.uk. The ethnicity of South Yorkshire is 95.0% White, 2.6% Asian, and 2.4% Black and many languages are spoken including Polish, Czech, Slovakian, Urdu, Punjabi, and Russian. For those patients unable to read English, an additional A4 sheet explains in 12 languages how patients can obtain the Health Questionnaire translated into their language.

A unique study number is assigned to each Health Questionnaire recipient. Each GP surgery stores a list of unique study numbers and the associated patient data (names; addresses; postcodes; NHS numbers; date of birth). Patient responses will be captured on patient completed Health Questionnaires either on paper or online.

### Routine data collected: Health Questionnaire (patient completed)

The South Yorkshire Cohort is collecting two types of data: patient completed data and NHS generated data. Patient generated data is collected by postal and internet self completed questionnaire. As there was no generic questionnaire which covered all the study questions, an A4 size 'Health Questionnaire' was designed specifically for this study (Additional File 3). In the first instance the Health Questionnaire is sent out to patients from recruited GP practices with the Invitation letter from their GP practice (Additional File 2). In the Health Questionnaire there are 31 questions addressing specific issues using single or multiple choice closed format questions. These questions are described on 6 sides of A4 using the headings: About you, Your health, Your exercise and food, Your health care, You and your education, You and your work. The origins of the Health Questionnaire questions are described in Additional File 4.

#### About you

On the first page, demographic information as to gender, date of birth, and how many children they have (under 18 years) is sought. Body Mass Index (BMI) is calculated from patient's self-reported height and weight. Information on waist to height ratio is a better predictor of chronic diseases than BMI for many patients [14], thus patients are also asked to report their waist measurement (a paper tape measure is included with the Health Questionnaire). Ethnicity is collected using the 17 Census 2001 categories in line with the Race Relations (Amendment) Act 2000 which requires public bodies to take account of race equality in policy making and service delivery. Patients are also asked *"Thinking about your own life and personal circumstances, how satisfied are you with your life as a whole?" *and asked to respond to a 10 point lickert scale (0 = Completely dissatisfied and 10 Completely satisfied).

#### Your health

Patients' health related quality of life' is assessed using the EuroQoL-5D (EQ-5D) [15] which is the most commonly used generic 'health related quality of life' instrument. Respondents are asked one question relating to the following five health dimensions: mobility, self-care, usual activities, pain/discomfort and anxiety/depression. The responses to these questions are used together with the associated preference-based algorithm [16] to generate a preference-based utility measure (anchored at 1 for full health and zero for death with negative values representing health states considered to be worse than death). The resulting index (range -0.59 to 1) is the preferred utility measure to generate quality adjusted life years in cost effectiveness analyses submitted to NICE in the UK. EQ-5D has been shown to be sensitive to the health related quality of life effects of obesity [17].

Patients are asked if they *"have any long-standing illness, health problem, condition or disability?" *and the following categories supplied: '*tiredness/fatigue', 'pain', 'insomnia', 'anxiety/nerves', 'depression', 'diabetes', 'breathing problems e.g. chronic bronchitis, asthma or emphysema', 'high blood pressure', 'heart disease', 'osteoarthritis', 'stroke', 'cancer', 'other'*. Patients are also asked about alcohol consumption in the last week and smoking status.

From an NHS perspective, as the main provider of medicines, there is a strong rationale for understanding more about use of medicines from an individual perspective and their relationship with patterns of obesity and patterns of other NHS resource use. This study will collect estimates of the amount of prescribed and over the counter medicines, including those that are used for the treatment of obesity (e.g. Orlistat), as well as those with weight gain side effects (e.g. oral steroids). Although a mean 15 prescriptions per person per annum are made in the UK community [18] there is little information on the effects of combining medications and thus their combined effect on obesity.

Patients are asked whether they are currently taking any medication and to provide information on both prescribed and self prescribed medication including name and strength of tablet, medicine, ointment, drops, inhaler or injection; whether it is prescribed or not; and what this is for. As no validated questionnaire for patient reported medication use was identified, an existing validated Medication Change Questionnaire [19] was adapted. The Medication Change Questionnaire [19] provides people with a simple structured form on which to record their medication use for seven days. In order to increase questionnaire response rates, the Medication Change Questionnaire has been modified by asking patients to record general daily use rather than use over 7 days.

#### Your exercise and food

Information on the extent to which respondents are physically active is collected using the following question from the Department of Health's General Practice Physical Activity Questionnaire [20]: A screening tool to assess adult physical activity levels, within primary care" *"During the last WEEK, how many hours did you spend on each of the following activities?: Physical exercise such as swimming, jogging, aerobics, football, tennis, gym, workout etc. Cycling, including cycling to work and during leisure time. Walking, including walking to work, shopping, for pleasure etc"*.

Patients are also asked *"Is managing your weight a concern for you?" *and to report whether they have ever used the following interventions to help manage their weight: increasing exercise, healthy eating, controlling your portion size, a slimming club, over the counter weight loss medication or meal replacements.

#### Your healthcare

In addition to the medication question, additional NHS and non NHS healthcare resource use is collected on hospital visits, GP visits and other healthcare visits (nurse, physiotherapist, dietician, midwife, mental health worker, psychotherapist, counsellors, care worker, social worker, health visitor, community health champion, health trainer and alternative therapist (acupuncturist, chiropractor, herbalist, homeopath, and osteopath).

#### You and your education

patients are asked which educational qualifications they have.

#### You and your work

In order to collect information on **socio-economic status**, the self-coded version of the National Statistics Socio-economic Classification (NS-SEC) [21] is included. This is a flexible and structured, occupation based classification but has rules to provide coverage of the whole adult population. Patients are also asked how many days off from work or households tasks or leisure activities they have taken as a result of ill health.

### Routine data collected: NHS generated data

For those patients who provide consent to access to their '*NHS health records (your prescriptions, diagnoses and GP/hospital visits)'*, NHS generated data on individual patients will be collected through searches of GP databases using local codes created for the study. Each GP clinical system will be searched in order to generate information on the following NHS generated data: age, sex, disease diagnoses associated with obesity (type II diabetes, chronic obstructive pulmonary disease, stroke, coronary heart disease, cancer, high blood pressure, and osteoarthritis), drug information, number of consultations in the last year, and number of referrals in the last year.

### Response rate

This study aims to maximise the response rate to the Health Questionnaires, in order to minimise differences between the study population and the reference (general) population. Based on reported response rates to previous postal questionnaires e.g. 60% [22], 66% [23] and 49% [24] and a 60% response rate to a Sheffield population based survey of 3,000 patients aged 18 - 95 [25], we anticipated a response rate of 45 - 50%.

### Research Governance and confidentiality

Research governance approval has been obtained from each of the four Primary Care Trusts (Sheffield, Barnsley, Rotherham, and Doncaster). Data will be held for 35 years (15 years beyond the proposed 20 year lifespan of the cohort). Data management will be provided by the University of Sheffield Clinical Trials Research Unit (CTRU) who adhere to their own Standard Operating Procedures (SOPs) relating to all aspects of data management including data protection and archiving.

### Data quality and data management

The CTRU uses the 'Prospect' data management system for the capture and storage of this data. Prospect stores all data in a PostgreSQL database on virtual servers hosted by Corporate Information and Computing Services at the University of Sheffield. Prospect uses industry standard techniques to provide security, including password authentication and encryption using SSL/TLS. Prospect uses a comprehensive privilege management feature to ensure authorised individuals have access to only the minimum amount of data required to complete their tasks, thereby restricting access to patients' personal identifiable data. Areas of the system used to manage personal or sensitive information require specific privileges to gain access.

In order to ensure the safety of this data it is encrypted using AES256. The NHS number is encrypted using a one way hashing function (SHA2). All other information, except the unique study number, is encrypted using the unhashed password (NHS number) as the key. When the participant logs in to the online survey system their password is used to unencrypt the data from the upload. This data is then used to pre-fill sections of the online entry form (but is not stored at this stage). Upon completion of each page of the form, the submitted data is then saved to the server.

The participant is only allowed to log in during a defined window (42 days from the date of upload). During this time the participant is permitted to edit the information they have submitted. Once this period has expired the data will be available to the study database users. For those patients who consent for the study team to have access, data held by their GP or Health Authority will be downloaded into electronic files and transferred to the University of Sheffield Clinical Trials Research Unit. The NHS Confidentiality Code of Practice (Nov 2003) [26] will be followed.

### Ethics approval and indemnity

NHS Research Ethics Committee approval for the South Yorkshire Cohort protocol was obtained on 27^th ^April, 2010 (REC ref: 09/H1306/97).

### Consent

A core feature of the cmRCT design is 'patient centred informed consent' i.e. patient information and consent aim to replicate that of real world routine healthcare rather than conform to the needs of standard trial designs. Therefore all cohort patients consent to provide observational data at the outset, be contacted again, and for their information to be used to look at the benefit of healthcare treatments; however, consent to "try" a particular intervention in the future is sought only from those offered that intervention. This method of obtaining consent replicates the 'patient centred' information and consent procedures that exist in routine health care, where clinicians provide patients with the information they need, at the time they need it. In this study if patients provide all the following three consents (A, B and C) they are then regarded as members of the South Yorkshire Cohort.

• *A: *Health Questionnaire data to be used to *'help the NHS improve the long term health of people living in South Yorkshire'*. Information relating to this consent is provided in the GP Invitation letter to their patients (Additional File 2). Consent to provide this data is implicit and signalled by return of the Health Questionnaire to the researchers.

• *B: Further contact from the researchers*. This information is provided in the Invitation letter from the GP (Additional File 2) and consent is explicitly sought on the back page of the Health Questionnaire (Additional File 3).

• *C: *The information that patients provide to be used *'to look at the benefit of health treatments'*. Information relating to this consent is provided in the Invitation letter from GPs and is explicitly sought on the back page of the Health Questionnaire.

In addition to consents A, B and C, supplementary consent (D) is sought for:

• *D: *Access to *'your health records'*. Information relating to this consent is provided in the GP Invitation letter and on the back page of the Health Questionnaire.

Consent to the possibility of random selection to any future trial treatment is not sought. We suggest that it is unethical to falsely raise expectations of trial treatments when these expectations will not be fulfilled for the majority of the South Yorkshire Cohort study population. For example, if in the future 5 randomised controlled trials are embedded within the South Yorkshire Cohort (with for example 400 patients randomly selected to the intervention arm in each trial), this would equate to 2,000 out of 20,000 patients randomly selected to try an intervention and 18,000 not selected.

### South Yorkshire Cohort Guardian

Lack of consent to randomisation from individuals is not an insurmountable ethical issue for cluster randomised controlled trials [27]. For cluster randomised controlled trials, it is argued that the role of a Guardian is key to their ethical conduct [28]. Leeds East NHS Research Ethics Committee has effectively taken on the role of guardian of the South Yorkshire Cohort. All studies embedded or nested within the South Yorkshire Cohort (including randomised controlled trial) are required to obtain approval from Leeds East Research Ethics Committee. This will help ensure the safety and rights of South Yorkshire Cohort members and manage the risk of some groups being 'over researched'.

### Analysis plan

In order to ascertain the extent and type of any non response bias at the population level, the responders and non responders will be compared with regards to their GP practice and their postcode, sex and age. Initial analyses will be descriptive. Three different types of dataset will be produced according to the type of consent given:

#### Survey

all completed returned Health Questionnaires (*Consent A*).

#### Longitudinal observational study

all completed returned Health Questionnaires (*Consent A*) with consent to be contacted again (*Consent B*).

#### Multiple Trials Facility

all completed returned Health Questionnaires (*Consent A*) with consent to be contacted again (*Consent B*) and consent for researchers to use the information they provide to look at the benefit of health treatments (*Consent C*).

For each dataset, analysis of key variables will be divided into six sections: height and weight will be used to calculate BMIs for all patients and for purposes of analysis patients will then be divided into six groups according to their reported BMI: underweight < 18.5, normal 18.5-24.9, overweight 25 -.29.9, obese 30 - 34.9, severely obese 35 - 39.9, morbidly obese 40 - 49.9.

The following types of NHS and non NHS healthcare resource use data will be analysed using descriptive statistics: medication (prescribed/self prescribed), hospital visits, contacts with other healthcare providers, and weight management strategies. Data will also be analysed in order to identify any interventions that may promote weight management. This will include cost effectiveness analysis and modelling of potential interventions (expected value of perfect information) to establish the potential need for further research. The burden of obesity in terms of economic costs will be estimated from a societal perspective. In order to make the results comparable with NICE guidelines [29], an NHS and Personal Social Services Prospective will also be undertaken, which will not include production loss. Resource use profiles will be used to calculate costs using national average unit costs from routine sources, e.g. NHS reference costs for inpatient resources [30]; Personal Social Services Research Unit costs of health and social care [31] such as GP visits, physiotherapy sessions. Costs of non-NHS interventions will be valued using market prices. Production losses will comprise lost employment and changes in employment as valued by the "New Earnings Survey". The EQ-5D will be analysed to provide estimates of Health Related Quality of Life.

Results will be compared with those reported in the following relevant studies [8, 10 and 25]. For those patients who consent to their NHS health records being searched then further analyses will be conducted using information on patients': chronic disease status: as recorded by GP including: diabetes, chronic obstructive pulmonary disease, stroke, coronary heart disease, cancer; NHS resource use: visits to NHS primary care healthcare professionals, visits to NHS secondary care.

### Study management

The South Yorkshire Cohort is hosted within the Obesity Theme of the NIHR CLAHRC for South Yorkshire [3]. The Obesity Theme Advisory Group includes obesity commissioners from each of the four South Yorkshire NHS Primary Care Trusts, service providers, researchers from both Sheffield universities, clinicians and the public from across the region. This group provides an initial scientific review function and performs a gatekeeper and signposting role for internal and external enquiries for access to South Yorkshire Cohort data.

*South Yorkshire Cohort Management Group *has responsibility for overseeing the management of the study including: monitoring study progress and advising on modifications required, oversight of the study conduct, strategic planning, planning grant applications, co-ordinating embedded study applications, oversight of the budget, ensuring the study meets the requirements of Research Governance and data protection, dissemination of results.

*South Yorkshire Cohort Operation Group *is responsible for the day to day implementation of the study, including co-ordination of the staff working on the project, recruitment of GPs and patients, data management, and adherence to the protocol.

### Intellectual property and principles of collaboration

The South Yorkshire Cohort is assembling and observing longitudinally a cohort of NHS patients which will provide the following benefits:

• ongoing population surveillance of adults of all ages

• information on the natural history of the condition(s) and any associated factors

• information on 'treatment as usual'

• long term outcomes.

• longitudinal observational data

• a representative population based sample of adults of all ages

• up to date information to facilitate evaluation as well as hypothesis generation

• a multiple trials facility for hypothesis testing

• a recruitment facility for qualitative and quantitative research studies

Researchers with proposals to conduct randomised controlled trials embedded within the South Yorkshire Cohort or longitudinal observational research or use cohort data should read the guidance and conditions for studies utilising the South Yorkshire Cohort research facility and then complete the outline proforma available on the South Yorkshire Cohort website [http://clahrc-sy.nihr.ac.uk/south-yorkshire-cohort.html]. Proposals will be reviewed by the South Yorkshire Cohort Management Group. The principles for agreeing a proposal and releasing data are that the proposal (a) meets the broad aims of the South Yorkshire Cohort study, (b) is scientifically rigorous and practically feasible.

## Discussion

This National Institute for Health research Collaborations for Leadership in Applied Health Research and Care for South Yorkshire funded study [3] is focussing on improving patient outcomes through the conduct and application of applied health research and service design in the areas of chronic long term conditions, self-management, health inequalities and public health [32]. The South Yorkshire Cohort uses the cmRCT design which combines the strengths of the standard observational, longitudinal cohort study design with a population based cohort facility for multiple randomised controlled trials in a range of long term health and weight related conditions (including obesity). This infrastructure will allow the rapid and cheap identification and recruitment of patients, and facilitate the provision of robust evidence to inform the management and self- management of health and weight.

## Competing interests

The authors declare that they have no competing interests.

## Authors' contributions

JN conceived the study. The writing of the original South Yorkshire Cohort protocol, the writing of this article, the design and piloting of the Health Questionnaire and the implementation of the protocol were all led by CR with the support of PB, CS, and JB. In addition a wide range of stakeholders were consulted on and contributed to both the South Yorkshire Cohort protocol and the Health Questionnaire including KT, CLC, AT, RC, CW, AL, TC, JN AM, GH, RM and TY. All authors read and approved this version of the manuscript.

## Pre-publication history

The pre-publication history for this paper can be accessed here:

http://www.biomedcentral.com/1471-2458/11/640/prepub

## Supplementary Material

Additional File 1 **Invitation to GPs letter**. Letter inviting GPs to participate in the study. Click here for file

Additional File 2 **GP Invitation letter to their patients**. Letter from GPs to their patients inviting them to participate in the study. Click here for file

Additional File 3 **Health Questionnaire**. Health Questionnaire (including consent form) sent to patients. Click here for file

Additional File 4 **Origins of Health Questionnaire questions**. The origins of the questions in the Health Questionnaire are described. Click here for file
