# Supplementary material for: South Yorkshire Cohort: a 'cohort trials facility' study of health and weight - Protocol for the recruitment phase
Source: BMC Public Health. 2011 Aug 11;11:640. doi: 10.1186/1471-2458-11-640 (PMC3175187; doi:10.1186/1471-2458-11-640)
Supplement: Additional File 1 — Invitation to GPs letter. Letter inviting GPs to participate in the study. [file 1471-2458-11-640-S1.DOC]

Dr Joanna Blackburn

Research and Development

Block 14

Medical Physics

Barnsley Hospital

Gawber Road

Barnsley

S75 2EP

(Practice manager name and address)

(Date)

Dear (name, address)

**South Yorkshire Cohort – Health Questionnaire**

You are one of 40 South Yorkshire GP practices who have been chosen to take part in the ‘South Yorkshire Cohort’ study [http://clahrc-sy.nihr.ac.uk/south-yorkshire-cohort.html]. This is a joint NHS National Institute for Health Research and University of Sheffield study which aims to provide the information needed to help the NHS make decisions regarding the management of obesity and long term health. The study plans to do this by collecting and analysing information on the weight & health of 20,000 adults from South Yorkshire over the next 20 years.

We hope that you are interested in helping us with this study. This will involve:

- random selection of a proportion of adults registered with your practice (16-85yrs)
- mailing out a letter explaining the study and inviting them to fill in the postal questionnaire (enclosed)
- patients who are interested will then fill in the questionnaire and return it to the researchers at the University of Sheffield (using a SAE)
- patients will be asked if they consent to (i) receive further questionnaires (no more than one a year) and/or (ii) consent for their routine NHS health records being searched regarding their disease status and NHS resource use
- it is possible that a very small number of patients (2.5%) may be randomly selected to be offered an obesity related treatment being trialled in the future, and an even smaller number may be selected to be interviewed (0.5%) by the study researchers.

**Obesity practice profile**

For all practices who participate we will be able to provide a detailed profile of the health & weight of normal weight, overweight, obese and morbidly obese patients registered with your practice, plus the types of services and treatments they are using to manage their weight. We will provide whatever administrative and/or technical support that you might require to identify patients and mail them out the invitation letter and the questionnaire. We are also offering the incentive of payment for your participation.

Thank you

*signature*

Dr Joanna Blackburn

Study manager ‘South Yorkshire Cohort’
