# Supplementary material for: South Yorkshire Cohort: a 'cohort trials facility' study of health and weight - Protocol for the recruitment phase
Source: BMC Public Health. 2011 Aug 11;11:640. doi: 10.1186/1471-2458-11-640 (PMC3175187; doi:10.1186/1471-2458-11-640)
Supplement: Additional File 2 — GP Invitation letter to their patients. Letter from GPs to their patients inviting them to participate in the study. [file 1471-2458-11-640-S2.DOC]

[Insert name]

[Insert Address]

Date

Dear [Insert Name]

You are one of 40,000 adults who have been chosen at random to take part in the South Yorkshire Cohort. This is an NHS research study looking at the long term health of adults in South Yorkshire. Please help the researchers by filling in the health questionnaire. Your answers will help the NHS improve the long term health of people living in South Yorkshire.

The Health Questionnaire will take about 5-10 minutes to complete. When you have filled it in, put it in the envelope and post it to the researchers at the University of Sheffield. **NO STAMP NEEDED**. You can also fill in the Health Questionnaire online by going to: [http://syc.shef.ac.uk](http://syc.shef.ac.uk/). Enter your Study number and NHS numbers (found on the label on the back page of the Health Questionnaire).

All your answers will be made anonymous and used only for research purposes. No information will be given to anyone else. If you agree, the researchers may send you further questionnaires (no more than one or two a year) and use your answers to look at the benefit of long term health treatments. Also, if you agree, the researchers will look at your NHS health records (your prescriptions, diagnoses, and GP/hospital visits). It is completely up to you whether you consent or not. You can withdraw your consent from the study at any time in the future. If you do not want the researchers to contact you again, then return the Questionnaire without filling it in.

If you have any questions about the Health Questionnaire or the South Yorkshire Cohort study, go to the website <http://clahrc-sy.nihr.ac.uk/south-yorkshire-cohort.html>. Or you can contact the researchers (Dr Clare Relton and Dr Joanna Blackburn), at the University of Sheffield, 30 Regent Street, Sheffield, S1 4 DA. Tel: 0114 222 0796

Yours sincerely

[Insert Dr Names ]
