# Supplementary material for: South Yorkshire Cohort: a 'cohort trials facility' study of health and weight - Protocol for the recruitment phase
Source: BMC Public Health. 2011 Aug 11;11:640. doi: 10.1186/1471-2458-11-640 (PMC3175187; doi:10.1186/1471-2458-11-640)
Supplement: Additional File 4 — Origins of Health Questionnaire questions. The origins of the questions in the Health Questionnaire are described. [file 1471-2458-11-640-S4.DOC]

**Additional File 4: Origins of questions included in the Health Questionnaire**

| **Section** | **Question** | **Origin of question** |
| --- | --- | --- |
| **About You**  (page 2) | Waist Measurement | Adapted from BMI calculator website.  http://www.bmi-calculator.net/waist-to-hip-ratio-calculator/  Picture created by Making Sense. |
|  | Ethnicity | Census 2001- England, Individual Form  Gypsy/traveller category from Professor Paul Bissell. |
|  | Life Satisfaction | Additional optional item from the Australian Quality of Life Personal Wellbeing Index (Adult version), International Wellbeing Group (2006). |
| **Your Health** (page 3) | Health Related Quality Of Life | EuroQol 5-D Health Questionnaire (Rabin & de Charro, 2001) |
|  | Long Standing Conditions | Adapted from the Census, 2001 – England, Individual Form Question to include (i) conditions commonly associated with obesity (diabetes, Chronic Obstructive Pulmonary Disease, stroke, Coronary Heart Disease, cancer) and (ii) conditions most commonly reported as impacting on quality of life (tiredness/fatigue, pain, insomnia, anxiety/nerves and depression). |
| **Your Health** (page 4) | Alcohol | Sheffield Health Survey, 2000 |
|  | Smoking | Sheffield Health Survey, 2000 |
|  | Medication | Adaptation of Medication Change Questionnaire (Paterson, 2004) by South Yorkshire Cohort team. |
| **Your Exercise and Food** (page 5) | Physical Activity | The first three questions from the Department of Health’s General Practice Physical Activity Questionnaire (GPPAQ), 2006 |
|  | Weight Management  Strategies | Devised by South Yorkshire Cohort team after wide consultation with healthcare providers and patients. |
| **Your Health Care** (page 6) | Healthcare Resource Use | Developed by the South Yorkshire Cohort team in consultation NHS commissioners and providers. |
| **You and Your Education** (page 6) | Educational Qualifications | Census 2001 – England, Household Form |
| **You and Your Work**  (page 7) | Current Employment | Developed by South Yorkshire Cohort team |
|  | Work Related | National Statistics Socio-economic Classification (NS-SEC) Questionnaire – self coded version |
|  | Work and Ill Health | Developed by South Yorkshire Cohort team |
|  | Ill Health and Daily Tasks | Developed by South Yorkshire Cohort team |
